# *Carpinus gigabracteatus*, a new species from southeast Yunnan, China

**DOI:** 10.3897/phytokeys.145.49488

**Published:** 2020-04-10

**Authors:** Zhiqiang Lu

**Affiliations:** 1 CAS Key Laboratory of Tropical Forest Ecology, Xishuangbanna Tropical Botanical Garden, Chinese Academy of Sciences, Mengla 666303, Yunnan, China Xishuangbanna Tropical Botanical Garden, Chinese Academy of Sciences Mengla China; 2 Center of Plant Ecology, Core Botanical Gardens, Chinese Academy of Sciences, Mengla 666303, Yunnan, China Core Botanical Gardens, Chinese Academy of Sciences Mengla China

**Keywords:** *Carpinus gigabracteatus*, large bract, new species

## Abstract

*Carpinus gigabracteatus* Z. Qiang Lu, a new hornbeam species from southeast Yunnan of China, is described and illustrated in this study. It possesses extremely large bracts and is closely related to *C. tsaiana* Hu and *C. tschonoskii* Maxim., based on the characters of large bract size and bracts without lobes at the base of inner margins. Furthermore, morphological comparison suggested it was distinctly different from *C. tschonoskii* by a series of characters from leaf, infructescence, bract and nutlet and from *C. tsaiana* by its leaf length to width ratio (1.4–2.0 vs. 2.0–2.4), lateral veins significantly impressed adaxially, number of lateral veins on each side of midvein (9–14 vs. 14–17), bract length (3.9–4.8 vs. 2.5–3.2 cm) and bract length to width ratio (2.3–3.1 vs. 1.5–2.1). Therefore, this hornbeam, based on only one population from southeast Yunnan, is here erected as a new species, named as *C. gigabracteatus*.

## Introduction

The hornbeam genus *Carpinus* L. is the largest genus in the subfamily Coryloideae of Betulaceae (Holstein and Weigend 2017; Li et al. 2018). To the present time, more than 40 species have been published (Hu 1964; Qi 1981; Liang and Zhao1991; Li and Skvortsov 1999; Tong et al. 2014; Holstein and Weigend 2017; Lu et al. 2017, 2018). Due to their peculiar and beautiful fruit cluster, some hornbeams are used as important ornamental plants (Fini and Ferrini 2011; Li et al. 2018). The bract characters of fruit clusters are also important evidence for species identification (Hu 1964; Li and Skvortsov 1999; Lu et al. 2017). According to the bract characters, three morphological groups are separated by bracts completely covering the nutlet, all bracts with conspicuous lobes at the base of inner margins and bracts without lobes or rarely with inconspicuous lobes at the base of inner margins, respectively (Li and Skvortsov 1999; Lu et al. 2017). In China, the last is the largest group, including about 26 species (Holstein and Weigend 2017; Lu et al. 2018), most of them being narrow endemics within China (Li and Skvortsov 1999). Bract size is the critical trait for distinguishing these species between each other (Hu 1964; Li and Skvortsov 1999). Almost all species in this group have bracts less than 3.2 × 1.3 cm. However, the present author found a hornbeam population during field surveys in southeast Yunnan with bracts without lobes at the base of inner margins, but with large bracts (3.9–4.8 × 1.4–2.0 cm) and these could not be ascribed to any described species. In addition, those hornbeams distributed in other regions, including *Carpinus betulus* L., *C. caroliniana* Walter, *C. faginea* Lindl., *C. laxiflora* (Siebold & Zucc.) Blume, *C. orientalis* Mill. and *C. tropicalis* (Donn.Sm.) Lundell, all have smaller bract size than this Yunnan population, which also distinctly differs in bract lobes at the base of inner margins and leaf characters (Hu 1964; Furlow 1987; Holstein and Weigend 2017). However, in China, within the morphological group possessing bracts without lobes at the base of inner margins, only *C. tsaiana* Hu has the same bract width but differs from the Yunnan population by bract length. *C. tschonoskii* Maxim. has similar bract length but with different bract width (Li and Skvortsov 1999). The present author, therefore, hypothesised that this morphologically different population from southeast Yunnan may represent a potential new hornbeam. In order to test this hypothesis, the present author carried out morphological comparisons with representatives of all hornbeams in China.

## Material and methods

### Field surveys and specimen examination

Multiple rounds of field surveys on hornbeams in southeast Yunnan were conducted in the years 2013–2019. At first, only one population was found with extremely large bracts in 2018, this being different from all described Chinese hornbeams by the large bract size. In 2019, the present author collected samples to characterise species morphology, habitat, distribution and conservation status. Voucher specimens were deposited as *Zhiqiang Lu 2019GY0801*–*Zhiqiang Lu 2019GY0802* (HITBC) and *Zhiqiang Lu 20189801*–*Zhiqiang Lu 20189804* (LZU). Specimens (including type specimens) of all related hornbeams in China (Li and Skvortsov 1999) were consulted through CHV and GBIF platforms. However, hornbeams with bracts whose nutlets are covered completely are excluded from the morphological analysis (Li and Skvortsov 1999; Holstein and Weigend 2017). All information from all the 115 specimens examined is listed in Table 1.

**Table 1.** Specimens preserved in herbarium used for morphological comparison.

Species name	Collector	Collection number	Collection site	Herbarium	No. of specimens
*C. gigabracteatus*	*Z.Q. Lu*	*2019GY0801–2019GY0802*	Wenshan, Yunnan	HITBC	4
	*Z.Q. Lu*	*20189801–20189804*	Wenshan, Yunnan	LZU	2
*C. chuniana*	*C.L. Tso*	*20872*	Ruyuan, Guangdong	HUH	1
*C. chingiana*	*Q.S. Zhao et al.*	*6980 (three duplicates)*	Muli, Sichuan	CDBI	3
*C. dayongina*	*K.W. Liu*	*33359*	Zhangjiajie, Hunan	CSFI	1
*C. fargesiana*	*Q. Li*	*77351*	Jinchuan, Sichuan	PE	1
*C. firmifolia*	*P.H. Yu*	*810*	Bijie, Guizhou	KUN	1
*C. hebestroma*	*Anonymous*	*118773*	Hualian, Taiwan	Tai	1
*C. henryana*	*W.Y. Chun*	*4173*	Liangsungkou, Hubei	PE	1
*C. insularis*	*K.M. Tam*	*0770924*	Hongkong	IBSC	1
*C. kawakamii*	*K. Taiya*	*1998*	Taiwan	Tai	1
*C. lipoensis*	*Y.K. Li*	*9940*	Libo, Guizhou	HGAS?	1
*C. luochengensis*	*J.Y. Liang*	*K1644 (two duplicates)*	Luocheng, Guizhou	IBK	2
*C. mengshanensis*	*F.Z. Zhao*	*84001*	Pingyi, Shandong	SDFS	1
*C. microphylla*	*Z.C. Chen*	*54089*	Tianyang, Guangxi	IBK	1
*C. mollicoma*	*K.M. Feng*	*1203*	Xichou, Yunnan	PE	1
	*Z.Q. Lu*	*201511501-201511517*	Xichou, Yunnan	LZU	17
*C. monbeigiana*	*H.R.E. von Handel-Mazzetti*	*3431*	Yunnan	K	1
	*Z.Q. Lu*	*2016WXYZ001- 019*	Weixi, Yunnan	LZU	19
*C. omeiensis*	*K.H. Yang*	*57490 (three duplicates)*	Emei, Sichuan	PE, NAS	3
*C. paohsingensis*	*T.H. Tu*	*4356 (two duplicates)*	Baoxing, Sichuan	PE	2
*C. polyneura*	*E.H. Wilson*	*5791*	Emei, Sichuan	HUH	1
*C. pubescens*	*A. Henry*	*9928 (two duplicates)*	Mile, Yunnan	PE, K	2
*C. purpurinervis*	*Y.K. Li*	*P01567 (five duplicates)*	Duan, Guangxi	IBK	5
*C. rupestris*	*J. Cavalerie, Z.S. Zhang*	*4560, 6624*	Guizhou	PE	2
*C. shensiensis*	*Y.Y. Pai*	*2860, 2891*	Shaanxi	PE	2
*C. shimenensis*	*P.C. Cai*	*20241*	Shimen, Hunan	CSFI	1
*C. turczaninovii*	*S.W. Williams*	*12681*	Beijing	GH	1
*C. tibetana*	*Z.Q. Lu*	*2016QTP001-011*	Bomi, Xizang	LZU	11
*C. kweichowensis*	*Y. Tsiang*	*4406*	Zhenfeng, Guizhou	PE	1
*C. viminea*	*N. Wallich*	*2800a (two duplicates)*	Nepal	K	2
*C. londoniana*	*A. Henry*	*11640*	Puer, Yunnan	K	1
*C. tientaiensis*	*Y.L. Keng*	*1065*	Tiantai, Zhejiang	PE	1
*C. putoensis*	*K.K. Tsoong*	*94 (two duplicates)*	Putuo, Zhejiang	PE	2
*C. langaoensis*	*Z.Q. Lu*	*2016LZQ029*	Langao, Shaanxi	LZU	1
*C. tschonoskii*	*M. Furuse*	*52662-52665, 52569, 12997*	Japan	PE	6
	*S. Tschonoski*	*s.n.*	Japan	PE	1
	*Sichuan team*	3759	Yuexi, Sichuan	PE	1
	*Y.X. He*	*23333*	Changhua, Zhejiang	HHBG	1
*C. tsaiana*	*H.T. Tsai*	*62398 (three duplicates)*	Pingbian, Yunnan	PE	3
	*C.W. Wang*	*85686 (four duplicates)*	Xichou, Yunnan	PE	4
	*Anonymous*	*217*	Huishui, Guizhou	GFS	1

### Morphological analysis

Comparative analyses of bract size for these related hornbeams were conducted. For the measurement of bract width, bract lobes were not calculated. Then, the closely related hornbeams, based on bract size, were selected from 33 hornbeam species. Furthermore, morphological differences of the Yunnan population were illustrated, based on a series of morphological characters from the leaf, infructescence, bract and nutlet. One to three representative bracts were chosen to conduct the measurement for each of the specimens. In addition, values of minimum and maximum bract width/length, recorded in *Flora of China* and other published studies (Hu 1964; Li and Skvortsov 1999; Tong et al. 2014; Holstein and Weigend 2017; Lu et al. 2017, 2018), were also used to determine the closely related species, based on the comparative analysis of bract size and other characters. Finally, many morphological differences between this Yunnan population and other closely related hornbeams were clarified through the morphological comparison, based on 115 specimens (including type specimens).

## Results

**Figure 1.**
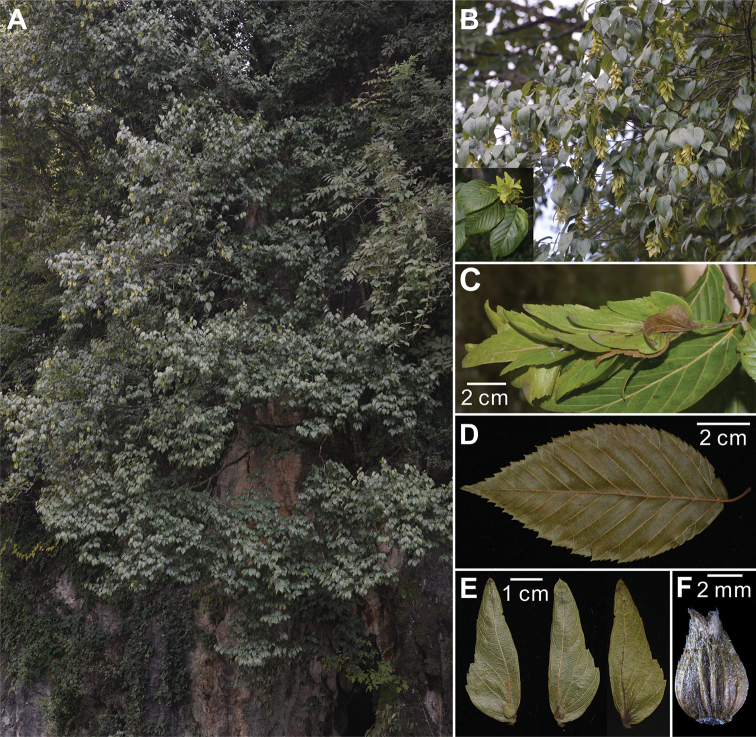
*Carpinus gigabracteatus* Z. Qiang Lu **A** the whole plant, habitat and location **B** branches with infructescences and leaves **C** infructescences **D** leaf **E** bracts **F** nutlet.

**Figure 2.**
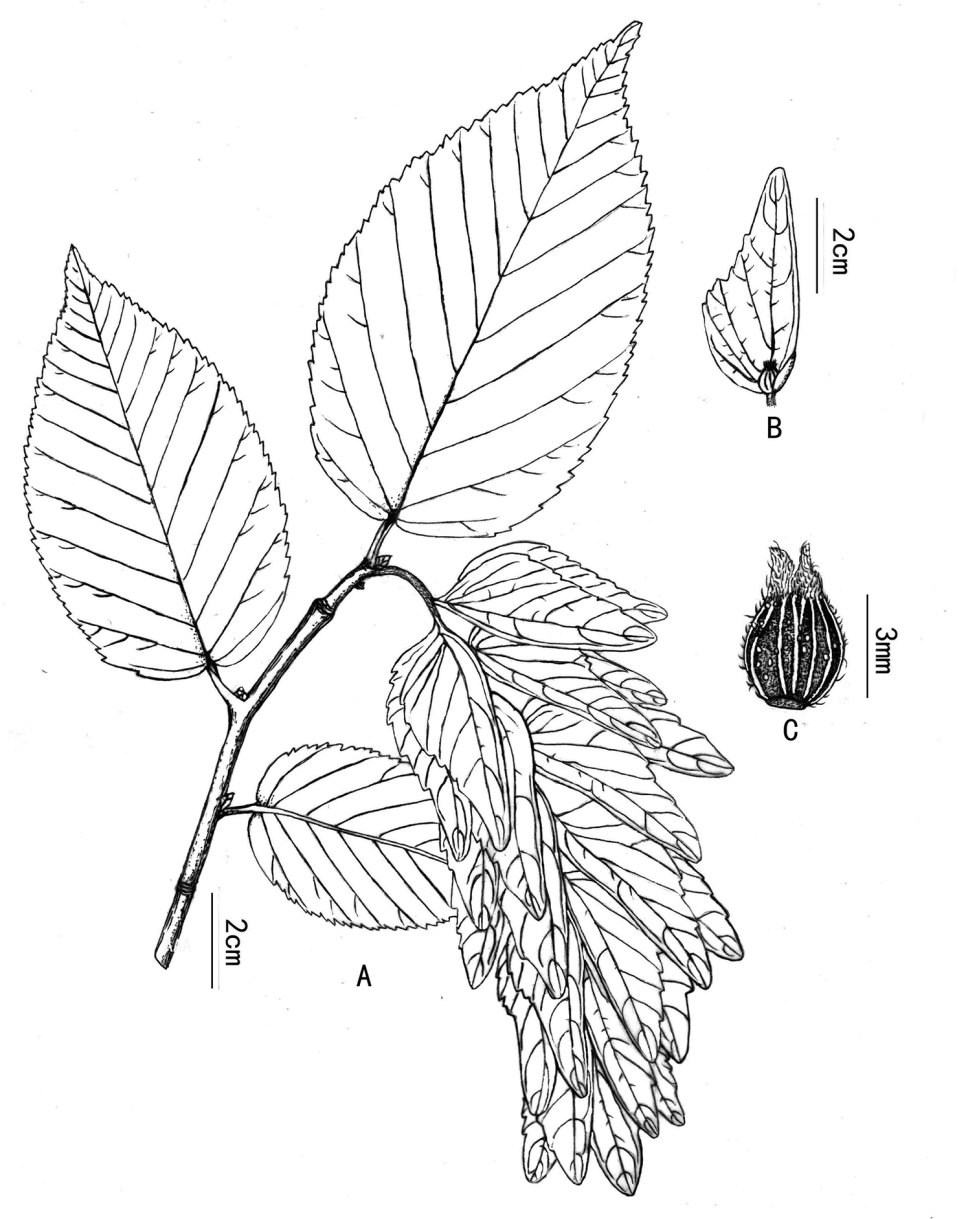
*Carpinus gigabracteatus* Z. Qiang Lu was drawn from *Z.Q. Lu 2019GY0801* (HITBC).

**Figure 3.**
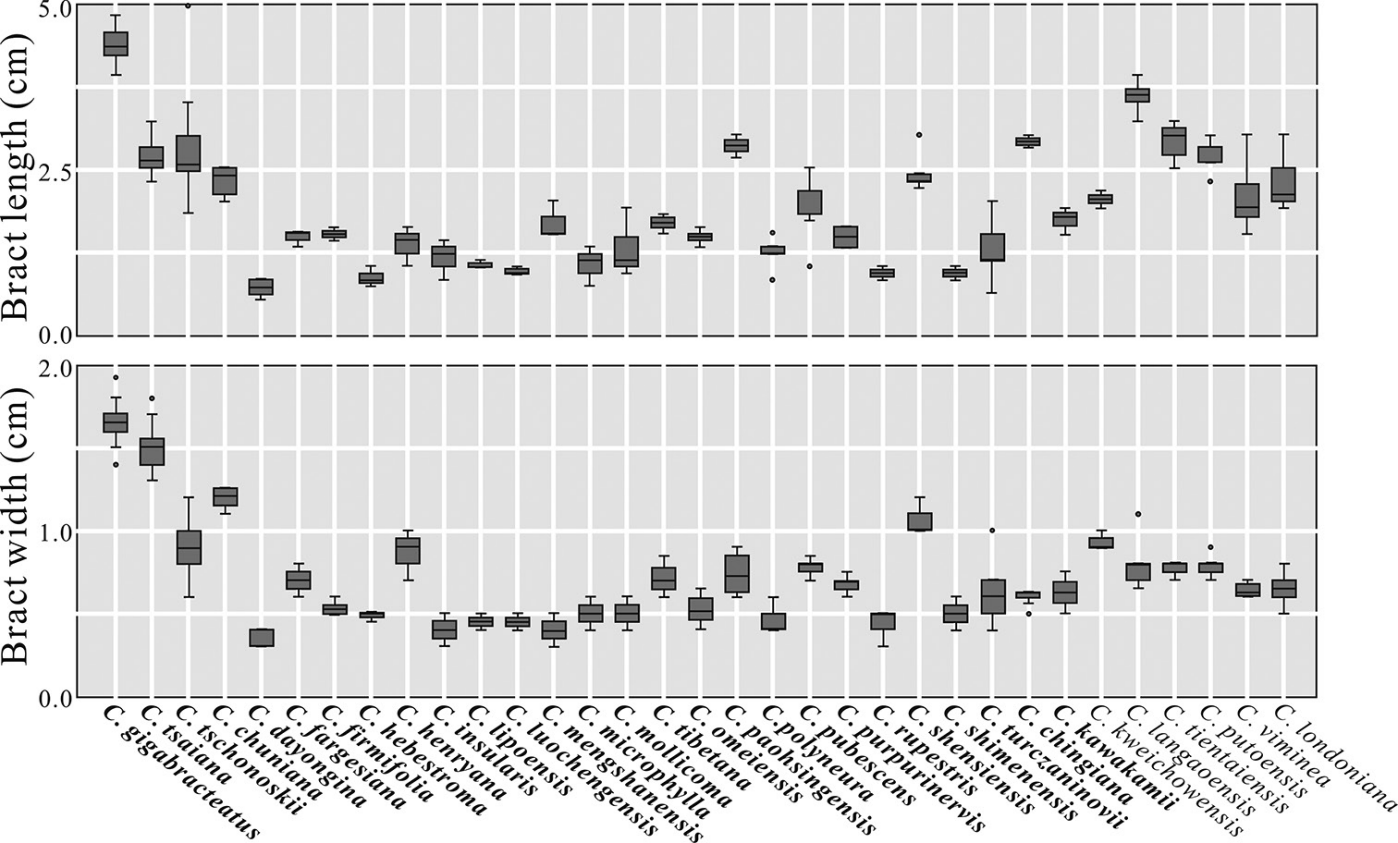
Phenotypic differentiation of bract length and width, across all Chinese hornbeam species according to Holstein and Weigend (2017). Data from all examined specimens in Table 1 and descriptions by Hu (1964), Qi (1981), Liang and Zhao (1991), Li and Skvortsov (1999), Tong et al. (2014) and Lu et al. (2017, 2018). Those hornbeams, whose bracts are without lobes or rarely with inconspicuous lobes at the base of inner margins, are in bold.

This hornbeam population from southeast Yunnan possesses extremely large bracts (3.9–4.8 × 1.4–2.0 cm) (Figures 1, 2). Phenotypic differentiation of bract length and width for hornbeams in China showed it was closely related to *C. langaoensis*, *C. tsaiana* and *C. tschonoskii* (Figure 3). Bracts, with and without lobes at the base of inner margins, corresponded to *C. langaoensis* and the Yunnan population, respectively (Figure 3). Morphological comparison with *C. tsaiana* and *C. tschonoskii* showed the Yunnan population distinctly differed from *C. tschonoskii* by leaf length to width ratio (1.4–2.0 vs. 2.0–2.3), lateral veins significantly impressed adaxially, infructescence size (8.0–12.0 × 5.0–5.5 cm vs. 6.0–10.0 × 3.0–4.0 cm), bract width (1.4–1.8 vs. 0.6–1.2 cm), nutlet shape (ovoid-ellipsoid vs. broadly ovoid), nutlet size (5.3–7.0 × 4.0–5.5 mm vs. 4.0–5.0 × 3.0–4.0 mm) and densely pubescent or villous and resinous glandular on nutlet (Table 2) and from *C. tsaiana* by leaf length to width ratio (1.4–2.0 vs. 2.0–2.4), lateral veins significantly impressed adaxially, number of lateral veins on each side of midvein (9–14 vs. 14–17), bract length (3.9–4.8 vs. 2.5–3.2 cm) and bract length to width ratio (2.3–3.1 vs. 1.5–2.1).

**Table 2.** Morphological comparison of *C. gigabracteatus* with *C. tsaiana* and *C. tschonoskii*.

**Characters**	***C. gigabracteatus***	***C. tsaiana***	***C. tschonoskii***
LEAF			
Shape and size	Leaf blade elliptic, ovate-elliptic or ovate, 7.0–12.0 × 4.0–7.0 cm, **length to width ratio 1.4–2.0**, base rounded, rounded-cuneate or cordate, margin regularly or irregularly and doubly minutely serrate, apex acuminate	Leaf blade elliptic, oblong, oblong-lanceolate or ovate-lanceolate, 8.0–14.0 × 4.0–7.0 cm, **length to width ratio 2.0–2.4**, base cordate or obliquely cordate, margin irregularly and doubly minutely serrate, apex acuminate	Leaf blade elliptic, oblong or ovate-lanceolate, 5.0–12.0 × 2.3–5.0 cm, **length to width ratio 2.0–2.3**, base subrounded or subrounded-cuneate, margin doubly setiform serrate, apex acuminate or caudate-acuminate
Length of petiole	7–14 mm	7–15 mm	7–15 mm
Number of lateral veins on each side of midvein	**9–14**	**14–17**	**12–16**
Lateral veins significantly impressed adaxially or not	**Significantly impressed adaxially**	**Not**	**Not**
Abaxially densely villous or sparsely villous along veins	Densely or sparsely villous	Sparsely villous	Sparsely villous
INFRUCTESCENCE			
Size of infructescence	8.0–12.0 × **5.0–5.5 cm**	10.0–15.0 × **4.0–5.5 cm**	6.0–10.0 × **3.0–4.0 cm**
Length of peduncle	1.5–2.5 cm	1.5–3 cm	1–4 cm
BRACT			
Size of bract	**3.9–4.8 × 1.4–1.8 cm**	**2.5–3.2 × 1.3–1.8 cm**	**1.8–5.0 × 0.6–1.2 cm**
Length to width ratio	**2.3–3.1**	**1.5–2.1**	**2.4–4.2**
NUTLET			
Shape and size of nutlet	**Ovoid-ellipsoid, 5.3–7.0 × 4.0–5.5 mm**	**Ovoid-ellipsoid, 5.0–6.0 × 4.5–5.0 mm**	**Broadly ovoid, 4.0–5.0 × 3.0–4.0 mm**
Densely pubescent or villous	**Densely pubescent, densely villous at apex**	**Densely pubescent, densely villous at apex**	**Glabrous except sparsely villous at apex**
Densely resinous glandular or not	**Densely resinous glandular**	**Densely resinous glandular**	**Usually no resinous glandular**

### Taxonomic treatment

#### Carpinus gigabracteatus

Taxon classificationPlantae

Z. Qiang Lu sp. nov.

1A9331E7-C13E-57E8-9DFA-4FD63DBF3C91

urn:lsid:ipni.org:names:77209333-1

Figures 1 , 2 大苞鹅耳枥

##### Diagnosis.

*Carpinus gigabracteatus* differs from *C. tsaiana* by leaf length to width ratio 1.4–2.0 (compared to 2.0–2.4), lateral veins impressed adaxially, 9–14 lateral veins on each side of the midvein (compared to 14–18), bract length 3.9–4.8 cm (compared to 2.5–3.0 cm) and bract length to width ratio 2.3–3.1 (compared to 1.5–2.1).

##### Type.

China. Yunnan: Wenshan Prefecture, 23°09'35"N, 104°05'53"E, 1591 m alt., karst limestone hill, 23 Sep 2019, *Z.Q. Lu 2019GY0801* (holotype, HITBC; isotypes, HITBC and LZU).

##### Description.

Tree to 8 m tall, deciduous; bark grey, smooth. Branchlets black-brown, glabrescent. Petiole 7–14 mm, densely yellow pubescent when young, glabrescent in the following few months; leaves alternate, leaf blade elliptic, ovate-elliptic or ovate, usually 7–12 × 4–7 cm, length to width ratio 1.4–2.0, leathery, abaxially sericeous-villous or sparsely villous along veins, bearded in axils of lateral veins, adaxially densely villous when young, base rounded, rounded-cuneate or cordate, margin regularly or irregularly and doubly minutely serrate, apex acuminate; lateral veins 9–14 on each side of midvein, raised abaxially, significantly impressed adaxially. Male inflorescence pendulous, spicate-cymose, cylindrical, enclosed by buds during winter, with many overlapping bracts, 1.0–3.0 × 0.4–0.6 cm when mature; flowers without bracteoles, inserted at base of bracts. Female inflorescence terminal or axillary on dwarf shoots, racemose; flowers paired; bracts leaf-like, complanate, overlapping. Mature infructescence 8.0–12.0 × 5.0–5.5 cm; peduncle 1.5–2.5 cm, densely yellow hirsute; giant bracts loosely overlapping, 3.9–4.8 × 1.4–1.8 cm, abaxially densely yellow hirsute along reticulate veins, outer margin coarsely dentate and rarely entire, commonly without but sometimes with basal lobe, inner margin entire, with inflexed basal auricle, apex acuminate; veins 5–6. Nutlet ovoid-ellipsoid, 5.3–7.0 × 4.0–5.5 mm, densely pubescent, densely villous at apex, densely brown resinous glandular, prominently 9 or 11-ribbed.

##### Etymology.

This hornbeam from southeast Yunnan has extremely large bracts, which are distinctly different from other closely related hornbeams, and therefore is given the epithet *gigabracteatus*.

##### Phenology.

Flowering from April to May and fruiting from July to September.

##### Habitat, distribution and conservation.

Up to now, only one *C. gigabracteatus* population has been collected from southeast Yunnan. For its population census, only six mature trees (6–8 m in height) and 13 seedlings grow on a steep karst limestone hill. To the present author’s knowledge, the bract size of this species is now the largest across the whole hornbeam genus in China. Hence, it has great horticultural and ornamental value and some people like to dig them up to grow them as ornamental trees. Manual digging involves removing lots of large rocks on the limestone hill where this new species grows, resulting in significant damage to the habitat. So far, no other population has been found, even though multiple rounds of field surveys in Wenshan Prefecture and adjacent regions have been carried out in the years from 2013 to 2019. Therefore, this hornbeam is exposed to significant threats from human activity due to its rarity and horticultural and ornamental value. According to the IUCN Categories and Criteria (IUCN 2016), the present author here classifies this species as “Critically Endangered” (CR). Fortunately, these mature trees can provide the possibility to expand population based on seeds.

##### Additional specimens examined.

China. Yunnan: Wenshan Prefecture, 23°09'35"N, 104°05'53"E, 1591 m alt., karst limestone hill, 23 Sep 2019, *Z.Q. Lu 2019GY0802*; the same locality, 10 July 2019, *Z.Q. Lu 20189801–Z.Q. Lu 20189804.*

## Discussion

Bract morphology in the *Carpinus* genus provides important traits for species identification (Hu 1964; Li and Skvortsov 1999; Lu et al. 2017, 2018). In this study, the present author demonstrated a hornbeam population from southeast Yunnan as a new species, based on the following evidence. First, its large bract size, including the characters of bract length and width, showed it to be closely related to *C. langaoensis*, *C. tsaiana* and *C. tschonoskii* (Figure 3). However, this Yunnan population, with its bract without lobes at the base of inner margins, can be easily distinguished from *C. langaoensis*, whose bracts have conspicuous lobes at the base of inner margins (Li and Skvortsov 1999; Lu et al. 2017, 2018). In addition, more characters, based on leaf and nutlet, can also distinguish both of them (Lu et al. 2017). Furthermore, other hornbeams distributed outside of China, including *C. betulus, C. caroliniana, C. faginea, C. laxiflora, C. orientalis* and *C. tropicalis*, are all different from this hornbeam population from southeast Yunnan, by the smaller bract size and other characters of bract and leaf (Hu 1964; Furlow 1987; Holstein and Weigend 2017). Finally, morphological comparison suggested it differed from *C. tschonoskii* by a series of characters from leaf, infruGctescence, bract and nutlet (Table 2), which was consistent with the description by Li and Skvortsov (1999). Therefore, the most similar species to the Yunnan population is *C. tsaiana*, based on similar morphology and distribution (Li and Skvortsov 1999; Holstein and Weigend 2017). However, all eight typical specimens of *C. tsaiana* (including seven type specimens) from three populations were distinctly different from this Yunnan population by leaf length to width ratio (1.4–2.0 vs. 2.0–2.4), lateral veins significantly impressed adaxially, number of lateral veins on each side of midvein (9–14 vs. 14–17), bract length (3.9–4.8 vs. 2.5–3.2 cm) and bract length to width ratio (2.3–3.1 vs. 1.5–2.1) (Table 2; Figures 1–3). Hence, the present author proposes to recognise this hornbeam population from Yunnan as a new species.

## Supplementary Material

XML Treatment for Carpinus gigabracteatus
